# Genetic copy number variants in myocardial infarction patients with hyperlipidemia

**DOI:** 10.1186/1471-2164-12-S3-S23

**Published:** 2011-11-30

**Authors:** Wei-Chung Shia, Tien-Hsiung Ku, Yu-Ming Tsao, Chien-Hsun Hsia, Yung-Ming Chang, Ching-Hui Huang, Yeh-Ching Chung, Shih-Lan Hsu, Kae-Woei Liang, Fang-Rong Hsu

**Affiliations:** 1 Department of Information Engineering and Computer Science, Feng Chia University, Taichung City, Taiwan; 2 Department of Anesthesia, Changhua Christian Hospital, Changhua County, Taiwan; 3 Department of Computer Science, National Chiao Tung University, Hsinchu, Taiwan; 4 Department of Cardiovascular Medicine, Changhua Christian Hospital, Changhua County, Taiwan; 5 Department of Computer Science, National Tsing Hua University, Hsinchu, Taiwan; 6 Department of Medical Education and Research, Taichung Veterans General Hospital, Taichung City, Taiwan; 7 Department of Cardiovascular Medicine, Taichung Veterans General Hospital, Taichung City, Taiwan; 8 Master's Program in Biomedical Informatics and Biomedical Engineering, Feng Chia University, Taichung City, Taiwan

## Abstract

**Background:**

Cardiovascular disease is the chief cause of death in Taiwan and many countries, of which myocardial infarction (MI) is the most serious condition. Hyperlipidemia appears to be a significant cause of myocardial infarction, because it causes atherosclerosis directly. In recent years, copy number variation (CNV) has been analyzed in genomewide association studies of complex diseases. In this study, CNV was analyzed in blood samples and SNP arrays from 31 myocardial infarction patients with hyperlipidemia.

**Results:**

We identified seven CNV regions that were associated significantly with hyperlipidemia and myocardial infarction in our patients through multistage analysis (P<0.001), at 1p21.3, 1q31.2 (*CDC73*), 1q42.2 (*DISC1*), 3p21.31 (*CDCP1*), 10q11.21 (*RET*) 12p12.3 (*PIK3C2G*) and 16q23.3 (*CDH13*), respectively. In particular, the CNV region at 10q11.21 was examined by quantitative real-time PCR, the results of which were consistent with microarray findings.

**Conclusions:**

Our preliminary results constitute an alternative method of evaluating the relationship between CNV regions and cardiovascular disease. These susceptibility CNV regions may be used as biomarkers for early-stage diagnosis of hyperlipidemia and myocardial infarction, rendering them valuable for further research and discussion.

## Background

Cardiovascular disease is the principal cause of death in Taiwan and many countries, of which myocardial infarction (MI) is the most serious condition. Low-density lipoprotein (LDL) cholesterol, high-density lipoprotein (HDL) cholesterol, and triglyceride (TG) levels in the blood are risk factors for cardiovascular disease. Elevated concentrations of total cholesterol (TC) and LDL in serum are associated with an increased risk of coronary heart disease [1]. Hyperlipidemia is a significant cause of MI, because it is characterized by high serum TC, LDL, and TGs and because its complex mechanisms affect progressive atherosclerosis [2].

In the past decade, genome-wide association studies (GWASs) on hyperlipidemia and myocardial infarction have examined gene expression and heredity in families and identified several candidate genes and SNPs. GWASs have linked SNPs to susceptibility to cardiovascular diseases, such as familial combined hyperlipidemia (FCHL) [3] and dyslipoproteinemia [4]. Many GWASs of cardiovascular disease have identified several loci; for example, the WTCCC study reported nine loci that were robustly associated with coronary artery disease, including 9p3l and 9p34 [5]. These loci were also discovered in South Korean [6] and Italian populations [7]. These results indicate that gene variation mediates FCHL and hyperlipidemia.

Of the sequence variations in the human genome, copy number variation (CNV) contributes directly to changes in gene expression through gene dosage effects. CNV is a variation in the DNA sequence and can affect the expression of nearby and distal genes, causing phenotypic differences. Stranger *et al.* analyzed the association of expression levels of 14,925 transcripts with SNPs and CNVs, noting that signals from these types of variation have little overlap [8]. Examining the genome for both variants might be an effective means of determining the causes of complex phenotypes and diseases in humans.

CNV regions have been estimated to cover 18% of the human genome [9]. Inherited CNVs underlie mendelian diseases, and some copy number (CN) variable genes are associated with human diseases, such as schizophrenia and autism [10,11]. Lanktree and Hegele suggest that rare and common CNVs contribute to the susceptibility to metabolic disease. A common CNV in *LPA*, encoding apolipoprotein(a), is the primary determinant of plasma lipoprotein(a) concentrations and a risk factor for atherosclerosis, and CNVs in *LDLR* mediate heterozygous familial hypercholesterolemia in patients [12]. CNVs are a significant source of genetic diversity, but their influence on phenotypic variability, including disease susceptibility, remains poorly understood. Their findings have encouraged further investigation of the relationship between CNVs and cardiovascular disease.

With regard to cardiovascular disease, several articles on copy number variation in hyperlipidemia patients or copy number variation in myocardial infarction patients have been reported, but there is no CNV research integrating both diseases. In this study, we try to discover novel suspect CNV region from myocardial infarction patients with hyperlipidemia. Study of disease specific CNV region of myocardial infarction patients with hyperlipidemia might help to find out the disease mechanism and might aid in disease prediction.

## Results

### Analysis of CNV regions

A total of seven loci disrupted by CNVs were found. These included five gains and two losses regions, and these were associated significantly with hyperlipidemia and myocardial infarction (*P*≤0.001). The shortest CNV range is 1935 bp, and the longest CNV range is 9811 bp. The average of CNV range we observed is 5260 bp. A summary for each CNV region is presented in Table 1.

**Table 1:** Significant CNV regions

Cytoband	Start position of CNV region (bp)	Type	Total aberrations	Avg. CN	Length (bps)	Gene
**1p21.3**	95164205	Gain	10	3.057	6757	
**1q31.2**	193213064	Loss	13	0.76	4631	CDC73
**1q42.2**	232154677	Gain	15	3.4	9552	DISC1
**3p21.31**	45217649	Loss	21	1.217	3985	CDCP1
**10q11.21**	43623658	Gain	19	3.142	1935	RET
**12p12.3**	18574942	Gain	11	3.155	4461	PIK3C2G
**16q23.3**	82706957	Gain	12	3.161	5706	CDH13

**Figure 1:**
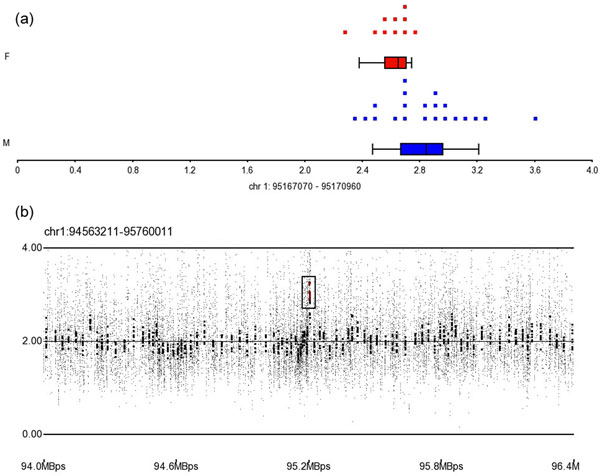
The CN state and SNP array profile graph of gain CNV region on lp21.3. (a) The distribution of CNs in the gain CNV region on 1p21.3. Blue and red dots indicate male and female patients, respectively; (b) SNP array profile for part of 1p21.3; the black box marks the location of the gain CNV region.

The gain CNV region on 1p21.3 (ch1: 95164205) lay near the start of solute carrier family 44, member 3 (*SLC44A3*)*.* This CNV region was detected in 10 patients of case group, and the average CN of this region is 3.06 (P=4.13×10^-3^). In NCBI dbSNP build 131, there were 79 SNPs lie in this region and 23 SNPs in these were reported by HapMap. The CN of each patients and average CN of this region were presented in figure 1a, and SNP array profile of this CNV region is presented in figure 1b.

**Figure 2:**
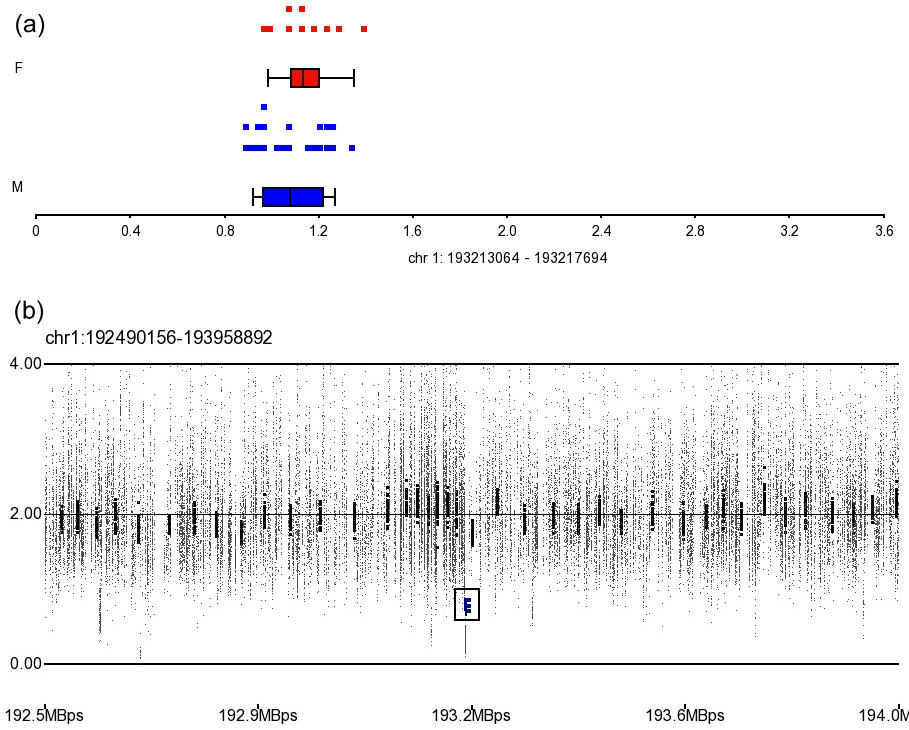
The CN state and SNP array profile graph of loss CNV region on 1q31.2. (a) The distribution of CNs in the loss CNV region on 1q31.2. Blue and red dots indicate male and female patients, respectively; (b) SNP array profile for part of 1q31.2; the black box marks the location of this CNV region.

The loss CNV region on 1q31.2 (ch1: 193213064) lay in the coding region of cell division cycle 73, Paf1/RNA polymerase II complex component (*CDC73*)*.* This CNV region was detected in 13 patients of case group, and the average CN of this region is 0.76 (P=2.29×10^-4^, Figure 2a and 2b). In NCBI dbSNP build 131, there were 28 SNPs lie in this region, but only rs7525286 and rs12117100 in these SNPs were reported by HapMap. We have not discovered any related literature to descript their role in hyperlipidemia or cardiovascular disease of these two SNPs.

The gain CNV region on 1q42 (ch1: 232154677) lay in disrupted in schizophrenia 1 (*DISC1*)*.* This CNV region was detected in 15 patients of case group, and the average CN of this region is 3.40 (P=3.5×10^-5^). *DISC1* encodes a protein with multiple coiled coil motifs that resides in the nucleus, cytoplasm, and mitochondria and regulates neurite outgrowth and cortical development through its interaction with other proteins. In NCBI dbSNP build 131, there were 109 SNPs lie in this region and 21 SNPs in these were reported by HapMap.

The loss CNV region on 3p21.31 (ch3: 45217649) lay in CUB domain containing protein 1 (*CDCP1*), which encodes a transmembrane protein that contains 3 extracellular CUB domains is expressed on the cell surface. This CNV region was detected in 21 patients of case group, and average CN of this region is 1.22 (P<1×10^-6^). In NCBI dbSNP build 131, there were 74 SNPs lie in this region, 11 SNPs in these were reported by HapMap.

**Figure 3:**
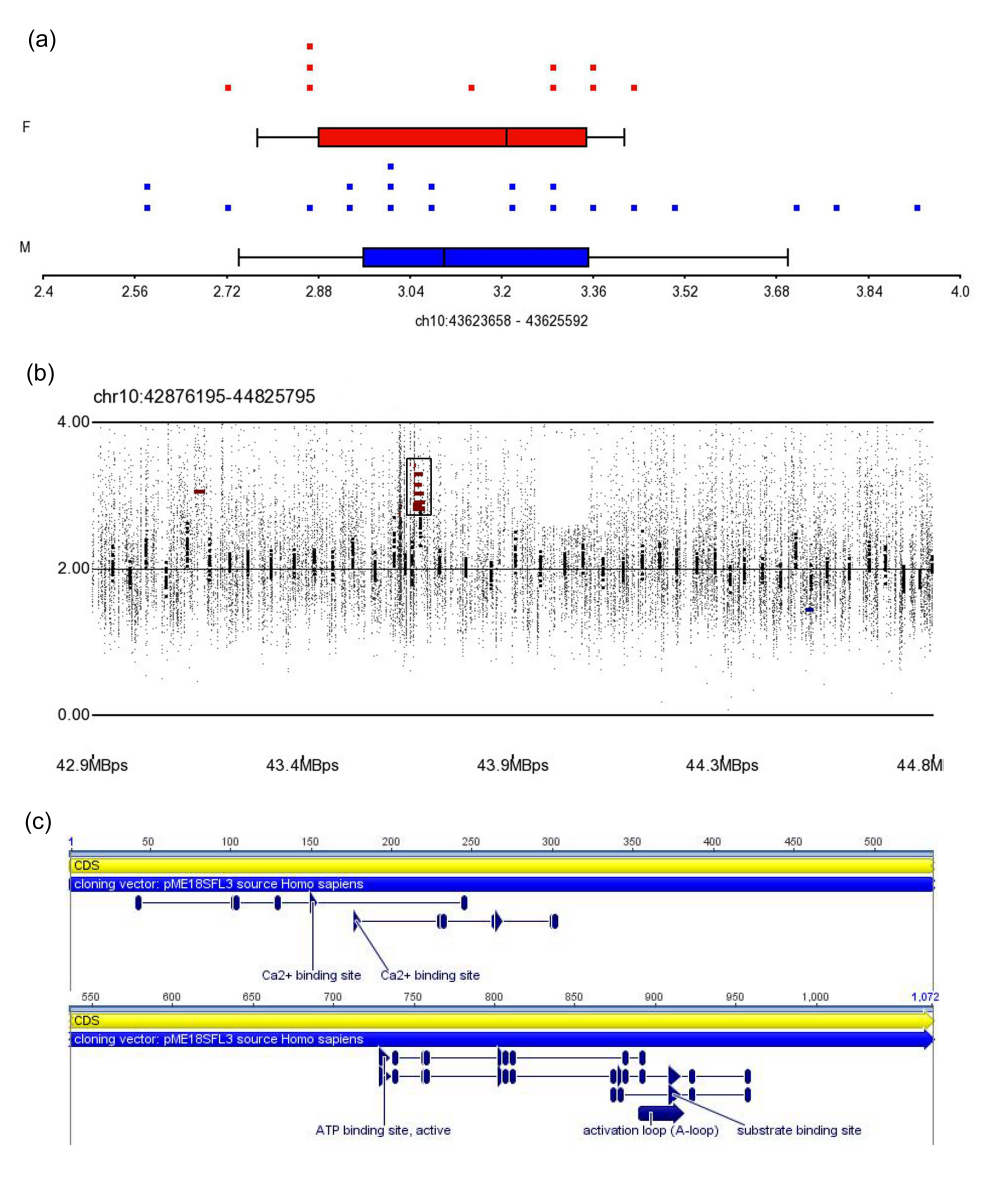
The CN state and SNP array profile graph of gain CNV region on 10q 11.21. (a) The distribution of CNs in the gain CNV region on 10q11.21. Blue and red dots indicate male and female patients, respectively; (b) SNP array profile for 10q11.21; the black box shows the location of this CNV region; (c) Characteristics and exon region of this gain CNV region in protein P07949.

The gain CNV region on 10q11 (ch10: 43623002) lay in the coding region of protein P07949 (Swiss-Prot: P07949.3) in ret proto-oncogene (*RET*)*. RET* is a member of the cadherin superfamily and encodes a receptor tyrosine kinase. This CNV region was detected in 19 patients of case group, and the average CN of this region is 3.14 (P=4×10^-6^, Figure 3a and 3b). There were six SNPs in this region that were reported by dbSNP build 132 and three SNPs in these (rs17028, re2742240, rs2435355) were reported by HapMap. We have not discovered any related literature to descript their role in hyperlipidemia or cardiovascular disease of these three SNPs unfortunately.

The gain CNV region in 12p12.3 (ch12: 18574942) lay in phosphoinositide-3-kinase, class 2, gamma polypeptide (*PIK3C2G*), which encodes a protein in the phosphoinositide 3-kinase (PI3K) family. PIK3C2G contains a lipid kinase domain and a C-terminal C2 domain, characteristic of class II PI3-kinases, and function of PIK3C2G has not yet been determined. This CNV region was detected in 11 patients of case group, and the average CN of this region is 3.15 (P=1.67×10^-4^). There were 42 SNPs lie in this region that NCBI dbSNP build 132 and 10 SNPs in these were reported by HapMap.

The gain CNV region in 16q23.3 (ch16: 82706957) lay in cadherin 13, H-cadherin, heart (*CDH13*)*.* Like *RET*, *CDH13* is a member of the cadherin superfamily, and it is a putative mediator of cell-cell interactions in the heart and negatively regulates neural cell growth. This CNV region was detected in 12 patients of case group, and the average CN of this region is 3.15 (P=5.51×10^-4^). There were 101 SNPs lie in this region that NCBI dbSNP build 132 and 20 SNPs in these were reported by HapMap.

### Verification of CNV region by real-time PCR

**Figure 4:**
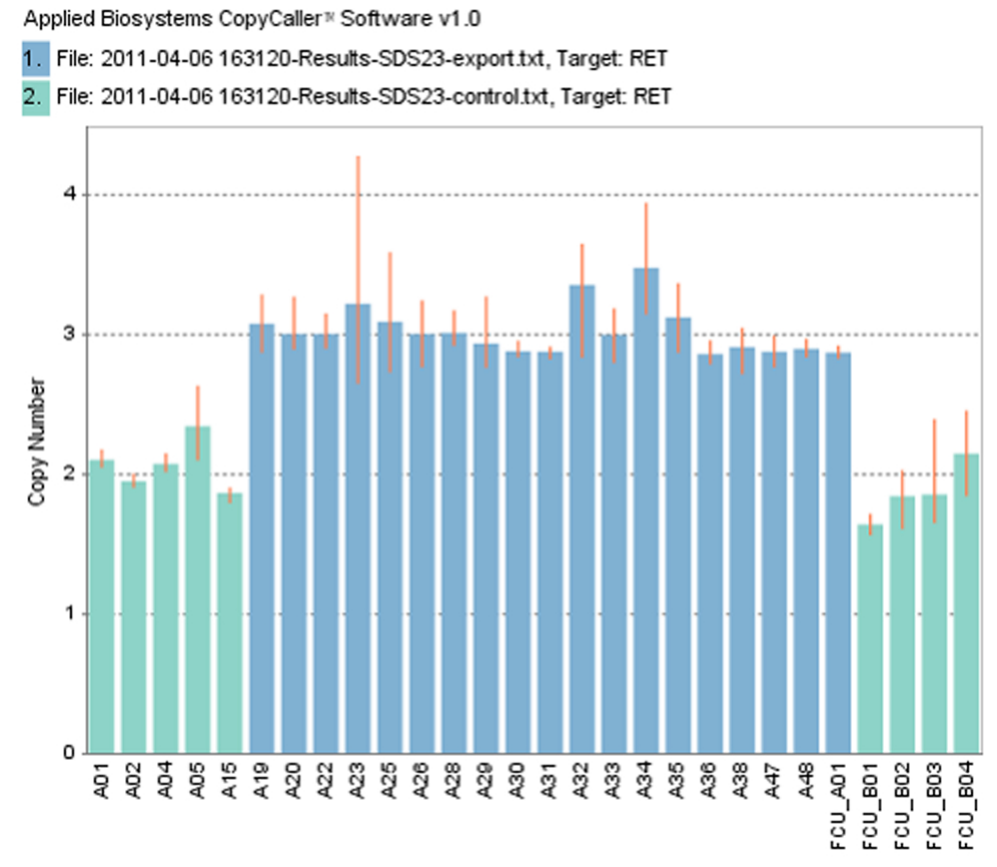
Real-time PCR result of the CNV region on 10q11.21 in *RET.* The CN state of case samples (n = 19, blue bar) are higher than the CN state of control samples (n = 9, green bar).

Figure 4 shows the real-time PCR results of the gain CNV region on 10q11.21 (ch10: 43623002). In 19 samples from case group which detected to have this gain CNV region, the average CN is 3.14235. Contrast the 9 samples from control group, the average CN is only 1.97 and shows the copy number of this region is unchanged. It confirms the real-time PCR experiment result is same to microarray.

## Discussion

CNVs have been implicated in few mendelian diseases on the molecular level. It would be premature to predict the relative proportion of complex diseases that are attributed to SNPs and CNVs due to the limited knowledge on the genetic basis of common, complex phenotypes [13]. In this study, hyperlipidemia and myocardial infarction-specific CNV regions were identified by CNV analysis on a SNP array, and some of which lay in regions that have been linked to hyperlipidemia and myocardial infarction in GWASs (Table 2). Yip *et al.* have linked atherogenic dyslipidemia and chromosome 1q31 [14]; Shmulewitz *et al. *[15] also observed that 1p31 and other regions are associated with significant heritability of metabolic traits. These regions coincide with our findings, and our CNV analysis narrows the range of functional gene regions.

**Table 2:** Description and related literature of identified CVNRs

Cytoband	Start position of CNV region (bp)	Gene	Literatures
**1p21.3**	95164205		
**1q31.2**	193213064	CDC73	Yip et al. [14] Shmulewitz et al. [15]
**1q42.2**	232154677	DISC1	Brouwers et al. [29]
**3p21.31**	45217649	CDCP1	
**10q11.21**	43623658	RET	Kiechl et al.[30] Samani et al.[5,31]
**12p12.3**	18574942,	PIK3C2G	Eijgelsheim et al.[32] Johnson et al.[33]
**16q23.3**	82706957	CDH13	Ruixing et al.[34] Avramoglu et al.[35]

In the present study, we have two interesting observations to bring out the possibility of relationship between the specific CNV region and hyperlipidemia in relative small p-value. The first observation is that the gain CNV region lies within the coding region of P07949 of *RET* (ch10: 43623002), and it is a member of the cadherin superfamily and encodes a receptor tyrosine kinase (Figure 3c). Chia implicated adhesion molecules in atherogenesis in a report on the relationship between cell surface proteins and hyperlipidemia and focusing on interactions between the endothelium, monocytes, and leukocytes and the influence of cytokines, oxidized low-density lipoproteins, and genetic determinants [16]. The second observation is that the gain CNV region on 16q23.3 (ch16: 82706957) lay in *CDH13.* Dong *et al.* noted the significant linkage between TC level and specific expression on 16q23 (LOD=3.35) in a genome-wide linkage scan of 1211 subjects from 100 Dominican families for 5 quantitative lipid traits, which identified several nearby genes, including *CDH13*[17]. The gene function of *RET* and *CDH13* is similar and belong to cell-surface protein, and several literatures bring out the function of these two genes may influence the level of low-density lipoproteins.

To further support our result, we have checked the haplotype data from our patient cohort. First, there are four haplotypes in represented CNV region of *RET* by query HapMap (rs7922745, rs10899917, rs17463850, rs1537796) and these four SNPs are not listed in the patient’s haplotype data. These four SNPs are located in the same block. Second, there is only a SNP rs8045610 in represented CNV region of *CDH13* by query HapMap, and this SNP is also not listed in the patient’s haplotype data. Although there is no direct evidence indicating a relation between hyperlipidemia and these two CNV regions, these regions were still valuable to investigate the role of regulation in related gene.

Comparative genomic hybridization (CGH) and single-nucleotide polymorphism (SNP) arrays are high-resolution tools that can be used to detect subtle CN alterations on the genomic scale. SNP arrays cost less and are easier to use than CGH in detecting one SNP and it can detect all SNPs in the entire genome, allele frequencies, and copy numbers in a single reaction. HMM segmentation and genomic segmentation are the principal algorithms in CNV detection and help us to discover the CNV in patients; however, multi-base and multi allele SNPs cannot be detected by SNP arrays and increase false negative and false positive. Additional experiment platforms such as fluorescence *in situ* hybridization (FISH), CGH array or real-time PCR will be needed to obtain more reliable results [13]. Fortunately, the real-time PCR result of observed CNV in 10q11.21 is same to microarray and in preliminary confirms our result.

As the sample size is small (n=31), we use the two-tailed Fisher's exact test and only consider events with high significance (P<0.001). Besides, these 31 patients are independent individuals; none of any two patients come from the same family. However, our findings may needs to be viewed with caution and be verified to step forward.

CNVs vary slightly in location and frequency between populations. The distribution of CNVs differs, based on genetic background, and population-specific CNVs can result in the divergence of physiological characteristics and disease prevalence between races [18]. Previous CNV discoveries were mostly limited to the sample from HapMap and to samples from Caucasian individuals, and other CNV survey studies using different ethnic backgrounds were needed. Therefore, all subjects in our study were Taiwanese and only Han Chinese and Japanese population data in HapMap project were considered for the help of CNV analysis.

Metabolic disorders were considered as correlated with multiple gene variation [19]. In this study, we discovered several CNV regions in patients and propose that might regulate gene expression and subsequent synthesis of specific proteins in hyperlipidemia, and we will make every effort to discover stronger evidences in the relationship between hyperlipidemia and specific CNVs in future.

## Conclusions

CNV is an important mechanism of hyperlipidaemia, and performing CNV analysis in hyperlipidemia and MI patients through bioinformatics methods is helpful in identifying disease-specific variants of genes. In this study, seven CNV regions that associated with hyperlipidemia and MI were discovered and real-time PCR was performed to confirm the result. After verified the independent of CNV and haplotype in patient cohort, these susceptibility CNVs reported in this study might associate with myocardial infarction patients with hyperlipidemia and might have the potential to be biomarkers.

## Methods

### Study subjects

Forty blood samples were collected from nine healthy Taiwanese controls and 31 Taiwanese patients from the department of cardiovascular medicine in Changhua Christian Hospital and Feng Chia University from December 11, 2009 to March 24, 2011.

The inclusion and exclusion criteria for enrolling patients were drawn from the Prospective Cardiovascular Munster study [20] and Framingham Heart Study [21]. In brief, patients aged between 20 and 75 years with coronary arterial disease with more than 50% stenosis, as proven by cardiac catheterization, and a history of hyperlipidemia with serum cholesterol level greater than 240 mg/dL or low-density lipoprotein greater than 155 mg/dL were enrolled.

The criteria for the control group included normal blood sugar concentration, normal blood cholesterol and lipoprotein concentration, normal electrocardiogram, no history of angina, normal cardiovascular physical examination results, and a 10-year risk of having a heart attack lower than 10% [22]. Participants with a history of diabetes, stroke, heart failure, or other major systemic diseases were excluded from the control group. The clinical information of the CAD patients and healthy controls are shown in Table 3.

**Table 3:** Clinical characteristics of patients and healthy controls

Variable	Affected (mean±SE)	Non-affected (mean±SE)
**N (subjects)**	31	9
**Age (years)**	45.0±18.3	36.8±17.2
**Gender (male/female)**	21/10	5/4
**Total cholesterol (mmol/L)**	265.7±10.5	-
**Triglycerides (mmol/L)**	244.9±34.4	-
**LDL cholesterol (mmol/L)**	164.0±9.6	-

Thirty one additional controls were selected randomly from 82 Han Chinese and Japanese samples of the International HapMap Project [23]. To minimize differences in gene variants between races, only Han Chinese and Japanese samples were selected for the control group to compare with the Taiwanese samples.

The data discussed in this publication have been deposited in NCBI's Gene Expression Omnibus (GEO) [24] and are accessible through GEO series accession number GSE31276. Ethical permission for all participants was obtained from the institutional review board of Changhua Christian Hospital, Taiwan.

### DNA extraction and preparation for microarray experiment

Genomic DNA was extracted from peripheral blood buffy coat using the Qiagen PureGene Blood Kit (Qiagen Inc., Valencia, USA) per the manufacturer's instructions. Before performing the microarray assay, the quality of the DNA was examined on a NanoDrop spectrophotometer (Thermo, Wilmington, USA). For each sample, 50 µg of genomic DNA was used to generate targets per the Affymetrix Genome-Wide Human SNP Array 6.0 protocol. Targets were prepared if 50 µg of amplified DNA was available and if they were between 250 and 2000 bp and hybridized per the manufacturer's recommendation.

### Detection of CNV

To identify all CNV regions that were linked to hyperlipidemia and myocardial infarction, multistage analysis was performed for 31 controls and 31 patients. First, all inbound SNP array data were imported after QC checked (Call Rate > 97%, Contrast QC > 0.4, MAPD < 0.3), and copy number profiles were created from allele intensities. The threshold parameter of normalization, quality control and outlier treatment of imported SNP arrays are following the manufacturer’s instruction. Population data from HapMap was used to generate the copy number (CN) baseline. Next, CNV detection was performed. The CN profile was analyzed using the genomic segmentation algorithm of PARTEK Genomic Suite 6.5 (PARTEK Inc., St. Louis, USA). The criteria with detected CNV segments were as follows: (1) neighboring regions with significantly different average intensities, and the significant level we chose is p-value less than 0.001, (2) breakpoints (region boundaries) that yielded the optimal statistical significance (smallest *p*-value), and (3) detected regions with at least 10 probes. Single nucleotide polymorphisms with a smoothing value below and above 2±0.3 were considered a loss and gain, respectively.

Next, we group all CNV regions in each sample which located in the same chromosome and have same start and stop position, and we get four values and a 2x2 contingency table for each CNV region: (a) patients with CNV, (b) controls with CNV, (c) patients without CNV, and (d) controls without CNV. Considering the small sample size, two-tailed Fisher’s exact test was performed to obtain a p-value for each CNV region, and the threshold of p-value we chose is 0.001.

Common CNV regions were excluded from these significant CNV regions using the Database of Genomic Variants (DGV) to specify the CNV regions in patients [25,26], NCBI RefSeq [27] and Geneious 5.4 [28] were used to annotate the location and coding region of each significant CNV region in the genome.

### Verification CNV region by real-time PCR

Quantitative real-time polymerase chain reaction (PCR) were performed using the TaqMan Copy Number Assay (Life Technologies Co., California, USA) to verify the CNV region on 10q11.21 (ch10: 43623658), the most significant result that we obtained (P<1×10^-5^). Nineteen patients and nine healthy controls were assayed for this CNV region by using the pre-designed probe Hs00975020_cn from the manufacturer. PCR was performed in quadruplicate; each 10-µl reaction comprised 10 ng gDNA and 1 TaqMan probe/primer mix in 1 TaqMan Universal Master Mix, amplified on an Applied Biosystems 7900HT SDS. The cycling conditions were 50°C for 2 min, 95°C for 10 min, and 40 cycles of 92°C for 15 sec, 60°C for 1 min. Real-time data were collected by the ABI SDS 2.3 software. ABI CopyCaller 1.0 software was used for real-time PCR data analysis.

## List of abbreviations used

CNV: copy number variation; SNP: single nucleotide polymorphism; array CGH: array-based comparative genomic hybridization; CNPs: copy number polymorphisms; MI: myocardial infarction; LDL: low-density lipoprotein cholesterol; HDL: high-density lipoprotein.

## Authors' contributions

WCS conceived of and designed the study; performed the molecular genetic studies; performed the SNP, CNV, and statistical analysis and drafted the manuscript. THK participated in CNV result analysis, coordinated blood sample collection from the patients, and helped draft and review the manuscript. YMT participated in all experiment methods design, performed real-time PCR experiments and DNA extraction. CHH, YMC, and CHH participated in the collection of patient blood samples and aided in the discussion of clinical causes and characteristics of hyperlipidemia. SLH and KWL provided crucial suggestions in the design of study and helped review the manuscript. YCC coordinated the resources and collaborators and help design the study. FRH participated in the design of this study and all discussions and helped review the manuscript.

## Competing interests

The authors declare that they have no competing interests.
